# Editorial: Immune Mechanisms in white matter lesions: Clinical and pathophysiological implications

**DOI:** 10.3389/fimmu.2023.1149625

**Published:** 2023-01-31

**Authors:** Xiao-Wei Pang, Cui Mei, Wei Qiu, Long-Jun Wu, Dai-Shi Tian

**Affiliations:** ^1^ Department of Neurology, Tongji Hospital, Tongji Medical College, Huazhong University of Science and Technology, Wuhan, China; ^2^ Department of Neurology, Huashan Hospital, Fudan University, Shanghai, China; ^3^ Department of Neurology, The Third Affiliated Hospital of Sun Yat-sen University, Guangzhou, China; ^4^ Department of Neurology, Mayo Clinic, Rochester, MN, United States

**Keywords:** white matter lesions (WMLs), immune mechanisms, microglia, ishcemic stroke, cerebral small vessel disease (CSVD), multiple sclerosis, neuromyelitis optica spectrum disorder (NMOsd)

White matter lesions (WMLs), often due to cerebral small vessel ischemia and several inflammatory demyelinated diseases such as multiple sclerosis (MS) and neuromyelitis optica spectrum disorder (NMOSD), are characterized as focal demyelination with a certain degree of inflammation. Brain-resident microglia and macrophages are highly dynamic cells that rapidly respond to cues in the injury sites and provide a neuroprotective or detrimental microenvironment for myelin maintenance by changing their activation state. Microglia/Macrophage phenotypic heterogeneity and their diverse responses are likely related to the differences in demyelinated pathology in WMLs. The peripheral immune cells, including attracted monocytes and T lymphocytes to the CNS, can also modulate microglia responses. The goal of this Research Topic is to gather contributions to advance research on immune mechanisms in white matter lesions as well as to explore potential interventions for ischemic white matter lesions or inflammatory demyelinated diseases.

## The critical role of microglia in WMLs

Microglia are the major immune cells of the central nervous system (CNS). The conventional view holds that the activation of microglia is deleterious in the pathological process, but accumulating evidence indicates that microglial activation may also have neuroprotective effects. The dual role of microglia suggests a diversity of potential functions of microglia (1). Specifically, in NMOSD, those demyelinated sites in WMLs are enriched with activated microglia and immersed macrophages, which together may contribute to neuro-inflammatory responses and neuronal damage. However, they also play protective roles by removing myelin debris, reducing inflammation, and secreting regenerative factors (Guerrero and Sicotte). Based on the recent understanding of microglial function, microglia-targeting therapeutic may represent a potential treatment for WMLs (Chen et al.).

## WMLs in ischemic stroke

Ischemic white matter lesions, the main pathological feature of cerebral small vessel disease (CSVD), is a disorder of cerebral microvessels that causes white matter injury, accompanied by inflammatory activation of microglia (2). Qin et al. found that white matter ischemic-induced microglial activation promotes the polarization of microglia towards a pro-inflammatory phenotype (3). In addition, rapid accumulation of autophagosomes was observed within activated microglia in chronic cerebral hypoperfusion. Accordingly, inhibition of microglial autophagy can shift the functional phenotype of microglia from pro-inflammatory to anti-inflammatory state, which may exhibit neuroprotective effects (4).

In this topic, Wang et al. assessed possible factors associated with WMLs heterogeneity in patients with cognitive impairment in CVSD. Wang et al. graded WMLs, enlarged perivascular spaces (ePVS), microbleeds, and lacunes on brain MRI. Cognitive impairment was assessed with Montreal Cognitive Assessment (MoCA) scores. Wang et al. defined mismatch as the severity of WMLs do not match the severity of cognitive impairment. Further, they used penetrating artery imaging to clarify this mismatch’s underlying mechanism. Eventually, Wang et al. suggested that conventional imaging features and penetrating artery damage may be responsible for the heterogeneity of WMLs in cognitively impaired patients with CSVD, which may be therapeutic targets for early identification and prevention of cognitive impairment Wang et al.

## WMLs in CNS inflammatory demyelinated diseases

### Multiple sclerosis

MS is the most common chronic inflammatory demyelinating disease of the CNS, which lead to immune-mediated inflammation, demyelination, and subsequent axonal damage in white matter (5). Traditionally, it has been thought that the pathological process in MS and mouse experimental autoimmune encephalomyelitis (EAE) models are mainly mediated through T cells. However, emerging evidence suggests that B cells and autoantibodies also play a considerable role in the disease process. In this topic, Seals et al. reviewed B cell biology and the role of B cells in autoimmune inflammatory demyelinating diseases and provided a novel review about the possible regulation of microglial activation by IgE in MS/EAE Seals et al.

In the early stages of MS, inflammatory cells infiltrate into the CNS through a compromised blood-brain barrier (BBB). Zhang et al. delineated the repulsion guide molecule-a (RGMa) is involved in the pathogenesis of MS/EAE by affecting BBB permeability. Next, Zhang et al. demonstrated that RGMa causes dysfunction of endothelial cells through BMP2/BMPR II/YAP, leading to disruption of BBB integrity in MS. This provides new insights for MS clinical treatment focusing on maintaining the BBB Zhang et al.

### Neuromyelitis optica spectrum disorder

NMOSD is an autoantibody-induced inflammatory disease of the CNS, which is mainly mediated by antiaquaporin-4 antibody (AQP4-ab) and involves the optic nerve and spinal cord. Previous studies have proved that microglial and macrophage activation are required for NMOSD pathogenesis Chen et al. Meanwhile, CD4^+^CD25^+^forkhead box P3^+^ (Foxp3) regulatory T cells (Tregs) play a central role in the immune regulation of NMOSD. Ma et al. discovered that Tregs attenuated immune cell infiltration in NMOSD mice, and polarized macrophages/microglia to an anti-inflammatory phenotype, thus mitigating white matter inflammation (6).

Besides, numerous B cell subsets also play significant roles in the pathogenesis of NMOSD. Wang et al. detected lymphocyte subsets in whole blood by flow cytometry and explored the changes in circulating lymphocyte subsets before and after immunotherapy for NMOSD and their correlation with clinical outcomes. Tacrolimus (TAC) inhibits the immune inflammatory response by interfering with the differentiation and proliferation of T cells and was used for the maintenance treatment of NMOSD in some studies. Wang et al. found that the proportions of some lymphocyte subsets changed obviously before and after TAC treatment. EDSS score may be associated with certain lymphocyte subsets after TAC therapy Wang et al..

In addition, predictors of disease relapses in AQP4-ab-positive NMOSD patients are critical for individualized therapy. Wang et al. developed an outcome prediction model and validated it in a multicenter validation cohort. Wang et al. reported a variety of factors, including demographics, clinical and therapeutic predictors of relapse, etc. to identify factors that predict relapse in AQP4-ab-positive NMOSD patients. These results suggest that early identification of patients at risk of adverse outcomes has significant implications for clinical treatment decisions Wang et al.

In summary, the Research Topic of “*Immune Mechanism in White Matter Lesions: Clinical and Pathophysiological Implications*” has collected a variety of valuable research and contributions on WMLs. The articles on this topic highlight the molecular mechanism involved in the pathology of demyelination and the innovative therapeutic approaches for white matter lesions, both of these are of clinical and pathophysiological importance.

## Author contributions

X-WP drafted the manuscript, CM, WQ, L-JWand D-ST collaborated the topics. All authors contributed to the article and approved the submitted version

## References

[1] Qin C Zhou LQ Ma XT Hu ZW Yang S Chen M. Dual functions of microglia in ischemic stroke. Neurosci Bull (2019) 35(5):921–33. doi: 10.1007/s12264-019-00388-3 PMC6754485 31062335

[2] Wardlaw JM Smith C Dichgans M. Small vessel disease: Mechanisms and clinical implications. Lancet Neurol (2019) 18(7):684–96. doi: 10.1016/S1474-4422(19)30079-1 31097385

[3] Qin C Fan WH Liu Q Shang K Murugan M Wu LJ. Fingolimod protects against ischemic white matter damage by modulating microglia toward M2 polarization *Via* Stat3 pathway. Stroke (2017) 48(12):3336–46. doi: 10.1161/STROKEAHA.117.018505 PMC5728178 29114096

[4] Qin C Liu Q Hu ZW Zhou LQ Shang K Bosco DB. Microglial Tlr4-dependent autophagy induces ischemic white matter damage *Via* Stat1/6 pathway. Theranostics (2018) 8(19):5434–51. doi: 10.7150/thno.27882 PMC6276098 30555556

[5] Karussis D. The diagnosis of multiple sclerosis and the various related demyelinating syndromes: A critical review. J Autoimmun (2014) 48-49:134–42. doi: 10.1016/j.jaut.2014.01.022 24524923

[6] Ma X Qin C Chen M Yu HH Chu YH Chen TJ. Regulatory T cells protect against brain damage by alleviating inflammatory response in neuromyelitis optica spectrum disorder. J Neuroinflamm (2021) 18(1):201. doi: 10.1186/s12974-021-02266-0 PMC8444427 34526069

